# 

**Published:** 1853-01

**Authors:** 


### CONTENTS

OF NO I.

### ORIGINAL COMMUNICATIONS.

### ESSAYS AND CASES.

ART. I.—History of the Medical Department of the University of Louisville: An Introductory Lecture, delivered Nov. 1st, 1852. By LUNSFORD P. YANDELL, M. D., Professor of Physiology and Pathological Anatomy. - - - - - 3

ART. II.—A Case of Infanticide. By W. L. SUTTON, M. D. of Georgetown, Ky. - - - - - - - - - - - 26

ART. III.—A Case of Ovariotomy. By S. D. GROSS, M. D. Professor of Surgery in the University of Louisville. - - 39

ART. IV.—Contributions to the History, Diagnosis, and Management of Croup. BY JOHN WARE, M. D., Professor of the Theory and Practice of Medicine in Harvard University. 45

### SELECTIONS FROM FOREIGN AND HOME JOURNALS

Hemorrhage from the extraction of a Tooth, - - - - - 63

Blood Changes in Disease, - - - - - - - - - - 64

The Medical Treatment of the late Duke of Wellington, - - 66

On the Use of Chloroform in Europe, - - - - - - 69
Murder committed by a Patient upon his Medical Adviser, - 74
Sudden Rupture of an Ovarian Tumor, - - - - - - 75
Obstruction of the Bowels, - - - - - - - - 78
On the Application of Friction, - - - - - - - 79
Surgical Sketches, - - - - - - - - - 82
New mode of taking Cod-Liver Oil. - - - - - - 86

ORIGINAL INTELLIGENCE.

A New Medical Journal. - - - - - - - - 87
Ricord's Letters on Syphilis, - - - - - - - 91
Mr. Webster's Brain, - - - - - - - - 92
Carpenter's Human Physiology - - - - - - - 92
Wythe's Pocket Dose Book - - - - - - - 93
Physicians Visiting List - - - - - - - - 93
Death of Dr. B. F. Wendel - - - - - - - 94

